# Supplementary material for: Validation of Non-Invasive Preimplantation Genetic Screening Using a Routine IVF Laboratory Workflow
Source: Biomedicines. 2022 Jun 11;10(6):1386. doi: 10.3390/biomedicines10061386 (PMC9219764; doi:10.3390/biomedicines10061386)
Supplement: Supplementary file 1 [file biomedicines-10-01386-s001.zip › biomedicines-1723412-supplementary.pdf]

Table S1. Clinical characteristics according to euploidy or aneuploidy in TE biopsy (PGT-A)

Variables	Euploidy, n=14	Aneuploidy, n=26	P
Age, yr	38.3 (2.8)	39.4 (1.7)	0.12
cfDNA amount in SCM, Qubit, ng/uL	2.11 (1.2)	1.53 (1.2)	**0.05**
Insemination method, IVF, n (%)	10 (71%)	16 (62%)	0.73
Arrest embryos, n (%)	3 (21%)	8 (31%)	0.72
Top-quality blastocyst[1], n (%)	2 (18%)	3 (17%)	1.00
**Timelapse morphokinetic time interval**			
Normal CC3, n (%)	11 (78.6%)	12 (46.2%)	**0.048***
Normal CC2, n (%)	11 (78.6%)	17 (65.4%)	0.385
Normal T5-T2, n (%)	11 (78.6%)	15 (57.7%)	0.187

Values are presented as Mean (SD) or n (%); SCM=spent culture medium; blastocyst[1]: >=3AA according to Gardner's grading. *=statistically significant.

Table S2. Validation with embryo section algorithm

Change selection priority, N (%)	No change selection priority, N (%)
**Model 1: If niPGT-A results were known in advance**	
7 (22.6%)	24 (77.4%)
**Model 2: The time-lapse morphokinetics were known in advance**	
6 (19.3%)	25 (80.6%)
**Model 3: niPGT-A then morphokinetics in advance**	
7 (22.6%)	24 (77.4%)
**Model 4: Morphokinetics then niPGT-A in advance**	
6 (19.3%)	25 (80.6%)

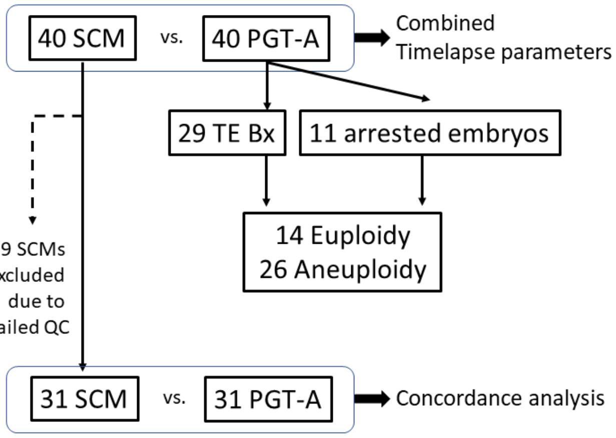

Figure S1. Study design of concordance analysis.

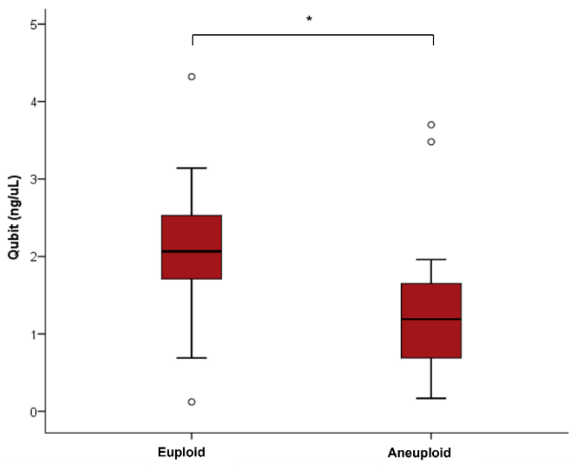

Figure S2. cfDNA amount in Euploid or Aneuploid embryos in maternal age >= 35 y/o. The ploidy here was according to TE/whole embryo PGT-A results. *=statistical significance.
